# Population Analysis of *Diospyros lotus* in the Northwestern Caucasus Based on Leaf Morphology and Multilocus DNA Markers

**DOI:** 10.3390/ijms23042192

**Published:** 2022-02-16

**Authors:** Lidia S. Samarina, Valentina I. Malyarovskaya, Ruslan S. Rakhmangulov, Natalia G. Koninskaya, Alexandra O. Matskiv, Ruset M. Shkhalakhova, Yuriy L. Orlov, Gregory A. Tsaturyan, Ekaterina S. Shurkina, Maya V. Gvasaliya, Alexandr S. Kuleshov, Alexey V. Ryndin

**Affiliations:** 1 Federal Research Centre “The Subtropical Scientific Centre” of the Russian Academy of Sciences, 354002 Sochi, Russia; q11111w2006@yandex.ru (L.S.S.); malyarovskaya@yandex.ru (V.I.M.); rakhmaruslan@yandex.ru (R.S.R.); natakoninskaya@mail.ru (N.G.K.); matskiv_a@mail.ru (A.O.M.); shhalahova1995@mail.ru (R.M.S.); grisha.tsaturyan@yandex.com (G.A.T.); shurkina-ekaterina@rambler.ru (E.S.S.); m.v.gvasaliya@mail.ru (M.V.G.); mister.alexandr.ru@gmail.com (A.S.K.); ryndin@vniisubtrop.ru (A.V.R.); 2 Federal Research Center N. I. Vavilov All-Russian Institute of Plant Genetic Resources (VIR), 190000 Saint Petersburg, Russia; 3 The Digital Health Institute, I.M. Sechenov First Moscow State Medical University of the Ministry of Health of the Russian Federation (Sechenov University), 119991 Moscow, Russia; 4 Agrarian and Technological Institute, Peoples’ Friendship University of Russia, 117198 Moscow, Russia

**Keywords:** *Diospyros*, cold tolerance, genetic diversity, ontogenetic strategy, leaf traits, ISSR, SCoT, polymorphism, morphological variability

## Abstract

*Diospyros lotus* is the one of the most frost-tolerant species in the *Diospyros* genera, used as a rootstock for colder regions. Natural populations of *D. lotus* have a fragmented character of distribution in the Northwestern Caucasus, one of the coldest regions of *Diospyros* cultivation. To predict the behavior of *D. lotus* populations in an extreme environment, it is necessary to investigate the intraspecific genetic diversity and phenotypic variability of populations in the colder regions. In this study, we analyzed five geographically distant populations of *D. lotus* according to 33 morphological leaf traits, and the most informative traits were established, namely, leaf length, leaf width, leaf index (leaf to length ratio) and the length of the fourth veins. Additionally, we evaluated the intraspecific genetic diversity of *D. lotus* using ISSR and SCoT markers and proposed a new parameter for the evaluation of genetic polymorphism among populations, in order to eliminate the effect of sample number. This new parameter is the relative genetic polymorphism, which is the ratio of polymorphism to the number of samples. Based on morphological and genetic data, the northernmost population from Shkhafit was phenotypically and genetically distant from the other populations. The correspondence between several morphological traits (leaf width, leaf length and first to fifth right vein angles) and several marker bands (SCoT5, SCoT7, SCoT30: 800–1500 bp; ISSR13, ISSR14, ISSR880: 500–1000 bp) were observed for the Shkhafit population. Unique SCoT and ISSR fragments can be used as markers for breeding purposes. The results provide a better understanding of adaptive mechanisms in *D. lotus* in extreme environments and will be important for the further expansion of the cultivation area for persimmon in colder regions.

## 1. Introduction

The Northwestern Caucasus is one of the northernmost growing regions for *Diospyros,* and this species was first introduced here between 1880 and 1890. Natural populations of *D. lotus* have a fragmented character of distribution in this region. *D. lotus* is the most cold-tolerant species in the genus *Diospyros* and is important for the development of new cold-tolerant varieties and is often used as a cold-tolerant rootstock for *D. kaki*, allow them to survive extreme temperatures down to −20 °C, with good yields [1,2]. This species is important as a genetic resource in breeding programs aimed at cold tolerance and early harvesting [3,4,5,6].

The adaptation of plant species to colder environment is believed to be due to phenotypic plasticity or adaptive evolution [7,8]. Phenotypic plasticity allows plants to adapt to the new specific environment [9,10,11,12,13] and it is an underexplored topic, although its understanding is crucial for predicting plant behavior in future climatic scenarios [13,14,15,16]. Comprehensive information on longitudinal patterns of morphological trait variation is very meaningful for exploring morphological diversity and evolutionary trends [12,17]. Under low-temperature stress, different morphological traits have a different character of variability [18,19,20,21]. General variability and the coefficient of determination are two important parameters for assessing morphological traits under stress [18,22]. These parameters are useful for revealing the level of morphological integrity of the trait under stressful conditions. Besides this, the index of vitality of coenopopulation (IVC) is one of the most important diagnostic characteristics of coenopopulations. It indicates the general status of populations and is used to count the ecological plasticity of the species [23]. These parameters help to evaluate the phenotypic plasticity of populations, reveal the mechanisms by which species acclimate in colder regions, and establish the most reliable morphological indicators of cold adaptation.

In small populations or fragments, selection for local adaptation is less efficient because of the effects of genetic drift on individual loci, and further, on the associations of alleles at different loci [24,25]. However, nobody knows whether most adaptations in trees are due to existing variation or new mutations. The population analysis of local naturalized species will help to reveal the mechanisms of cold adaptation and will allow us to better understand the evolutionary potential of the species.

Several DNA marker systems were developed and used for population studies in *Diospyros*, such as RAPD [26], SSRs [27,28,29,30,31], ITS-sequence [32], SRAP [29,33], IRAP [34], SCoT [34,35,36], ISSR [37] or orgDNA [38]. Among them, inter simple sequence repeats (ISSRs) and start codon targeted (SCoT) are efficient, reproducible and highly polymorphic multilocus markers for genetic diversity analyses [39,40]. The high occurrence of ISSRs between normal coding genes and their presence in certain chromosomes as satellite bodies, make ISSRs unique and advantageous for use in DNA fingerprinting. SCoT (start codon targeted) markers are based on polymorphism in the short, conserved region in plant genes surrounding the ATG translation initiation codon. The region flanking the ATG start codon is highly conserved in all plant species [41,42]. Thus, ISSR and SCoT markers can be useful to evaluate the genetic diversity within naturalized populations of *Diospyros lotus*.

The aim of this study was to analyze the variability among the geographically distant populations of *D. lotus* according to their morphological traits and multilocus markers, in order to predict ontogenetic strategy for *D. lotus* in the Northwestern Caucasus. The obtained results provide a better understanding of the acclimation strategy of *D. lotus* and will be important for the further expansion of the cultivation area for persimmon in the colder regions.

## 2. Results

### 2.1. Morphological Variability, Trait Determination and Ontogenetic Strategy of D. lotus Populations

**Figure 1:**
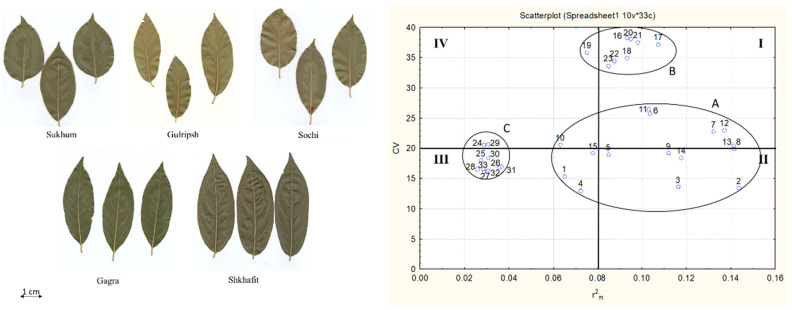
The character of variability of leaf traits in *D. lotus* populations. Abscissa—the coefficient of determination ($R_{m}^{2}$) ordinate—general variability (CV). I—Bio-ecological indicators (length of first and second veins, distance between veins); II—Biological indicators (leaf length, leaf width, leaf index, length of the fourth veins); III—Genotype-specific indicators (petiole length, leaf blade index, length of the fifth right vein, the angles of the second, third, fourth and fifth veins); IV—Ecological indicators (length of the fifth left vein, angles of the first veins, distance between fourth and fifth left veins); A—vein length, B—vein distances, C—vein angles. 1—petiole length, 2—leaf length, 3—leaf width, 4—LBI, 5—LI, 6–15—vein lengths, 16–23—distances between vein bases, 24–33—vein angles; the oval is the area of the dispersion of values.

A low level of general variability was observed for several traits: leaf length, leaf width, petiole length, Leaf Index (LI), Leaf Blade Index (LBI), length of the fourth veins, length of the fifth right vein, the angles of second, third, fourth and fifth veins. The greatest general variability was observed in the following traits: the length of the first and second veins, the distance between the bases of the first, second, third, fourth and fifth veins. The low coefficient of determination was observed for the following traits: the angle of the first, second, third, fourth and fifth veins, the length of the petiole and the LBI. Based on these data, all morphological traits were specified to one of the four groups of indicators with different characters of morphological variability (Figure 1):

I. Bio-ecological indicators—traits with a high CV and high $R_{m}^{2}$: length of first and second veins, distance between veins.
II. Biological indicators –traits with a low CV and high $R_{m}^{2}$. These are the most reliable indicators representing the general state of the plant organism under the current environment. These indicators are the leaf length, leaf width, LI and the length of the fourth veins.
III. Genotype-specific indicators –traits with a low CV and low $R_{m}^{2}$: petiole length, LBI, length of the fifth right vein, and the angles of the second, third, fourth and fifth veins.
IV. Ecological indicators—traits with a high CV and low $R_{m}^{2}$, for which variability is weakly consistent with the plant organism: length of the fifth left vein, the angles of the first veins, and the distance between the fourth and fifth left veins.

**Figure 2:**
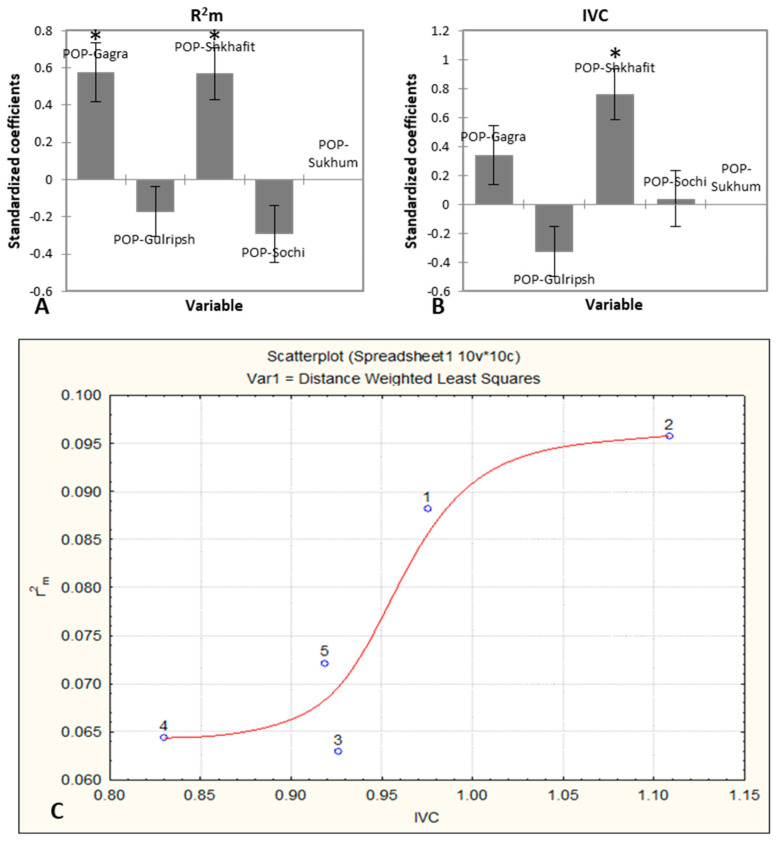
(**A**,**B**) The statistical significance of the differences in the coefficient of determination ($R_{m}^{2}$) and vitality index (IVC) between *D. lotus* populations, assessed by ANOVA: four s standardized over Sukhum, which is taken as 0; * represents significant differences at the confidence interval of 95%. (**C**) The trend of ontogenetic strategy of *D. lotus* on the Northwestern Caucasus. Each data point is calculated on about 10 trees (48–50 leaves per tree) and represents average values of $R_{m}^{2}$ and IVC, combined over 33 morphological traits: 1—Gagra, 2—Shkhafit, 3—Sochi, 4—Gulripsh and 5—Sukhum.

By analyzing the intraspecific differences, a significantly higher $R_{m}^{2}$ was detected in the Shkhafit and Gagra populations as compared to the other three populations (Figure 2A; Supplementary Table S1). In addition, the Shkhafit population showed a significantly higher vitality index (IVC), as compared to the other four populations (Figure 2B; Supplementary Table S1). Based on the intraspecific morphological diversity, the joint trend of the ontogenetic strategy of *D. lotus* was constructed. Two populations (Gagra and Shkhafit) showed higher IVC and $R_{m}^{2}$, as compared with the other three populations. However, the joint trend showed a depressive-type curve, indicating the weaknesses of the morphological integrity of *D. lotus* under increasing environmental stresses (Figure 2C).

### 2.2. Efficiency of ISSR and SCoT Markers, Intraspecific Diversity of D. lotus and Correspondence with Morphological Traits

Generally, the SCoT markers showed significantly higher discriminative power and higher PIC values than the ISSR markers for the intraspecific diversity analysis of *D. lotus*. The level of intraspecific genetic diversity was consistent between two marker systems: 0.50 (by ISSR data) and 0.47 (by SCoT data) (Table 1).

A total of 39 bands were detected with four ISSRs, ranging from Na = 7 (ISSR13) to 13 (ISSR880). The average number of polymorphic bands was 36.80%, ranging from 14.29% (for ISSR13) to 50.00% (for ISSR815) (Table 1). The average PIC was 0.34 and without significant differences among the ISSR markers.

A total of 52 bands were detected with six SCoTs, ranging from 4 (for SCoT07) to 13 (for SCoT20). The average number of polymorphic bands was 31.68%, ranging from 10.00% (for SCoT05) to 50.00% (for SCoT32). The mean PIC was 0.41, ranging from 0.38 (for SCoT05) to 0.46 (for SCoT07).

Generally, the ISSR markers detected a higher percentage of monomorphic bands, as compared to the SCoT markers. According to the SCoT data, Shkhafit and Gagra showed lower genetic polymorphism, as compared to the Gulripsh, Sochi and Sukhum populations (Table 2). Interestingly, the ISSR data did not correspond with the SCoT results, and showed the highest relative polymorphism in the Gagra populations, whereas the lowest relative polymorphism was revealed in the Gulripsh and Shkhafit populations (Table 2).

**Figure 3:**
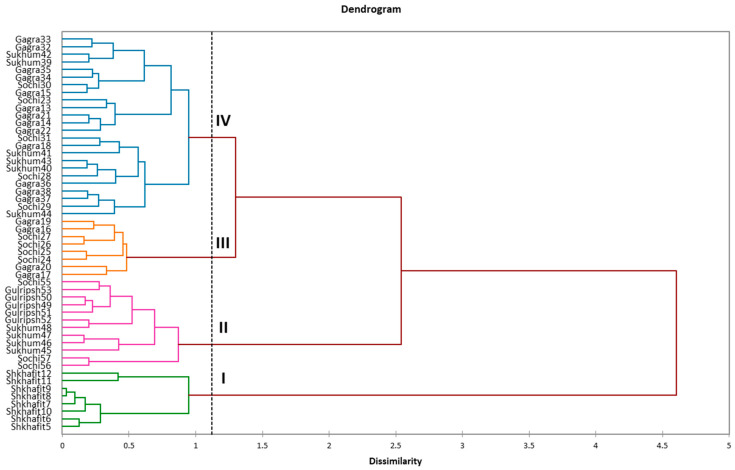
Genetic dissimilarities among the five *D. lotus* populations, calculated by the DICE method, based on ISSR and SCoT markers data.

The hierarchical clustering of 52 accessions based on SCoT and ISSR data, indicated four distant sub-branches (Figure 3). The biggest genetic distance was observed between the northernmost Shkhafit population (sub-branch I) and the other four populations. The second most distant sub-branch, sub-branch II, combined 12 accessions, which mostly belonged to the Gulripsh and Sukhum populations. In addition, sub-branches III and IV showed less genetic distance and were placed closer together. Wherein, sub-branch III consisted of eight accessions, which belonged to the Sochi and Gagra populations. Finally, the most abundant sub-branch, sub-branch IV, combined 24 accessions, which mostly belonged to the Sochi, Gagra and Sukhum populations. Thus, applied multilocus DNA-markers showed clear genetic dissimilarity between some geographically distant populations of *D. lotus*.

**Figure 4:**
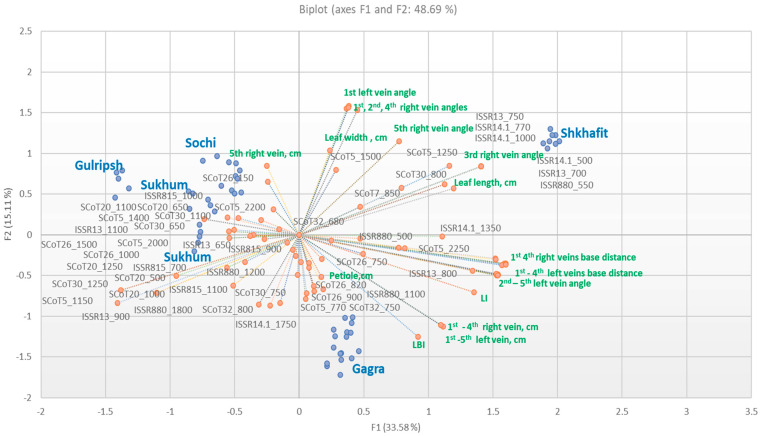
PCA biplot of the morphological and genetic variables of *D. lotus* populations: blue points—trees from five populations, orange points—DNA bands (bp) and morphological traits.

To detect a possible correlation and correspondence between genetic and morphological data, we used PCA biplot, representing the associated characteristics and relationships of variables (DNA bands, morphological traits) and observations (populations of *D. lotus*) (Figure 4). The first two PC showed 48.69% cumulative variation. The PCA biplot displayed the distribution of the accessions in several distinct groups: Shkhafit, Gagra, Gulripsh and the mixed group of Sochi and Sukhum populations.

According to PCA biplot, the accessions of Shkhafit were positioned on the positive sides of PC1 and PC2, with high loading. On the other hand, the accessions of Gagra were distributed on the positive side of PC1 and the negative sides of PC2. The accessions of Gulripsh, Sukhum and Sochi were grouped mostly on the positive loading of PC2 and the negative loading of PC1. In general, our previous results on relative genetic polymorphism among populations (Table 2) corresponded with the placement of the accessions on the PCA diagram: the widest spread of the data points were observed in populations with the greatest relative genetic polymorphism. Opposite to this, the densely grouped data points (the Shkhafit population) indicate the low level of relative genetic polymorphism.

Most vectors of the morphological characteristics were distributed with high loading located on the positive side of the PC1. The proximity of the accessions to the vectors was in agreement with the strong influence of those characteristics (vectors) on the closest accessions. For example, the Shkhafit accessions were placed closely to the vectors of several morphological traits (leaf length, leaf width, first to fifth right vein angles) and several marker bands (SCoT5_1250, SCot5_1500, SCoT7_850, SCoT30_800, ISSR14.1_500, ISSR14.1_770, ISSR14.1_1000, ISSR13_700, ISSR13_750, ISSR880_500 and ISSR880_550), which corresponded to the highest leaf length and width and to bigger vein angles in this population, as compared with the other populations. On the other hand, morphological traits, such as the first to fourth veins base distances, the length of leaf veins, left vein angles and LI, as well as some marker bands (SCoT5_2250 and ISSR13_800), highly correlated to each other and were positioned with high positive loading in PC1. Interestingly, the length of the fifth right vein was loaded on the orthogonal position, together with the Sukhum, Gulripsh and Sochi data points. In addition, several ISSR and SCoT bands showed close relationships to these accessions; however, their PC scores were low. Finally, many DNA bands were positioned as outliers on the negative side of both PCs, indicating neither an association with a certain population, nor an association with a certain trait.

## 3. Discussion

### 3.1. Morphological Variability, Trait Determination and Ontogenetic Strategy of D. lotus Populations

Morphological variation is commonly influenced by genetic variation, environmental variation, or the interactions between them [43,44,45,46]. Morphological variation underlies phenotypic plasticity, which is always present among plant leaves due to the modularity of their design, such that individual leaves can acclimate to their own environment [13,21]. In our study on *D. lotus*, different leaf traits were characterized by different characters of variability, indicating the different mechanisms underlying this variability, which is consistent with some other studies on several crops [12,13,17]. According to our results, we classified leaf morphological traits into four groups (I-bio-ecological indicators, II-biological indicators, III-genotype-specific indicators and IV-ecological indicators), based on correlation analysis. Compared with the other indicators, the biological indicators are considered as the most informative and highly determinative traits (high $R_{m}^{2}$ value), with a low level of general variability (low CV value). These indicators in *D. lotus* are leaf length, leaf width, Leaf index, and the length of the fourth leaf veins. Other studies on oak also showed that inter-population differences in leaf size were associated with the temperature at the sites of origin, suggesting local adaptation in response to diverging climates [9]. However, in another species, leaf width and leaf diameter did not correlate with climate, longitude or other environmental factors [12]. Probably, in different plant species, different morphological indicators underlie the adaptation and plasticity of this species.

Generally, a depressive type of ontogenetic strategy was established for species of *D. lotus*. However, among populations, the different trends of ontogenetic strategies indicate the better state of plants in the Shkhafit and Gagra populations, as compared to those from Sochi, Sukhum and Gulripsh. The index of vitality of coenopopulations represents the state of these populations in the current, and the ratio of vitality indexes over time indicates the ecological plasticity of the coenopopulations [23]. Interestingly, a significant difference in IVC was observed between Shkhafit and other populations. The higher IVC in the northernmost Shkhafit population showed the greater adaptive potential of this population. Furthermore, the higher $R_{m}^{2}$ value observed in the Shkhafit and Gagra populations indicates a higher level of morphological integrity of the morphological traits, and a better adaptability of these two populations in a changing environment.

### 3.2. Efficiency of ISSR and SCoT Markers, Intraspecific Diversity of D. lotus and Correspondence with Morphological Traits

In our study, SCoT markers displayed greater polymorphism and discriminative power as compared with ISSRs. This is consistent with Gorji et al. (2011), who also demonstrated that the SCoT technique was more efficient than the ISSR technique [47] for persimmon genotyping. SCoT markers are considered as “molecular marker genes” because they associated with start codons ATG and amplify the sequence between two different genes, and so could provide more information for breeding [2,34,36,48]. On the other hand, some studies on different plant species showed that ISSR markers were always more polymorphic than SCoTs [42,49,50,51]. However, other studies reported the better polymorphism of SCoT markers, compared to ISSRs [52,53,54]. We speculate that SCoT markers show better results than ISSR when analyzing homogenous sample sets with a low level of genetic diversity, as seen in the current research on *D. lotus*. On the other hand, ISSRs show better results than SCoTs when analyzing a diverse germplasm. This speculation is in accordance with recently published results on *Aegilops tauschii* [55].

The level of genetic diversity among different regions could reflect the ability of species to adapt to the environment to some extent [3]. Generally, we observed a low level of intraspecific genetic diversity in *D. lotus*; many ISSR and SCoT bands were monomorphic. On the other hand, other researchers detected more than 90% genetic polymorphism in *D. lotus* [2,34,36]. They reported an abundant phenotypic and genetic diversity among different *D. lotus* varieties worldwide. The low level of genetic diversity could be a constraint to the further expansion of the cultivation area of *D. lotus* in North Caucasus. Thus, introduction of more *D. lotus* germplasm from other sources is necessary in order to increase heterozygosity in local populations.

The main difficulty in comparing the level of polymorphism between studies and populations, is the different number of samples included in the different studies. A higher number of accessions usually shows a higher polymorphism in the sample set [52,53]. In this regard, we propose to use the relative level of polymorphism, which is the ratio between the polymorphism and the sample number. This will provide a better comparison of the genetic polymorphism level between different studies, or between different populations in one study.

Interestingly, according to genetic analysis, most accessions of the southernmost populations (Sukhum and Gulripsh) were placed on the greatest distance from the northernmost Shkhafit population. Along with this, it was indicated that the northernmost population from Shkhafit had the lowest level of genetic polymorphism. In contrast, the highest level of relative genetic polymorphism was detected in the southernmost Gulripsh population. Gulripsh, Sukhum and Gagra are the first locations of *Diospyros* introduction to the Western Caucasus [4,5], and this could be the cause of the higher genetic diversity in these locations. Genetic dissimilarity analysis showed mixed clusters IV and III, where the Sochi accessions were closely related to the Gagra and Sukhum accessions. We suppose that from there, this crop was transferred to Sochi and propagated. Lately, this germplasm moved to Shkhafit, expanding the cultivation area in a northern direction. In addition, the lower polymorphism, lower genetic diversity and close genetic relationships of the Shkhafit accessions, represented in Table 2 and Figure 3 and Figure 4, led us to the conclusion that this population was descended from a single ancestor, which was genetically and morphologically different from the other four populations. Based on the obtained results, we can speculate that adaptations to colder environments in *D. lotus* could be due to both existing trait variation and new mutations, as supported by several other studies [43,44].

Using PCA, we expected that the established biological indicators (leaf length, leaf width, Leaf index and the length of the fourth leaf veins) would be strongly associated within the Shkhafit population. Interestingly, among these biological indicators, leaf length and leaf width were closely associated within Shkhafit population. In addition, the first to the fifth right vein angles were positively loaded with the Shkhafit data points. Vein angles had low CV and low $R_{m}^{2}$ values and were classified as genotype-specific indicators, which are not subjected to any variability (Figure 1). On the other hand, leaf length and leaf width were proposed as the most important morphological indicators, as confirmed by PCA. Additionally, the PCA results indicate that most of the other leaf traits were significantly correlated with each other, which is consistent with some other studies [12,13]. Most bio-ecological and ecological indicators (with high CV values) were placed closer in the PCA biplot. Interestingly, genotype-specific indicators, such as petiole length, LBI and fifth right vein length, showed a distant position from them. Thus, PCA only partly supported our classification of morphological indicators into the four distinct groups. Finally, the unique bands associated with the Shkhafit data points could be used as important markers for further genetic diversity studies in *Diospyros*.

## 4. Materials and Methods

### 4.1. Plant Material and Morphological Evaluations

**Figure 5:**
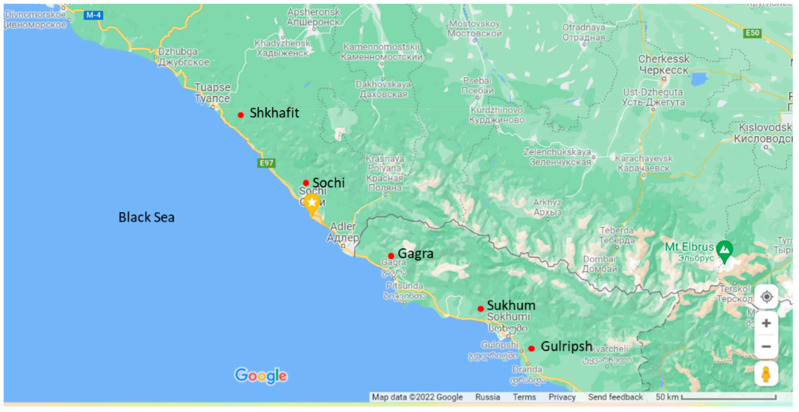
Geographical distribution of five *D. lotus* populations (red circles) included in genetic analysis.

For morphological analysis, 52 trees of five populations with 48–50 leaves per tree were evaluated. All trees were fully developed, about 10–18 m high and 50–80 years old. The distances between the trees within each population were 50–600 m. The distances between populations were about 30–50 km. In total, 33 morphological leaf traits were evaluated (Supplementary Table S1; Figure 5). The minimum winter temperatures for each population were negative 3 ± 2 °C (Gulripsh, Sukhum), negative 5 ± 2 °C (Gagra), negative 7 ± 2 °C (Sochi), and negative 13 ± 2 °C (Shkhafit).

Scanning of the leaves was performed during the day using the scanner HP PhotoSmart (600 × 600 dpi in JPEG). ImageJ [54] software was used for the assessment of the leaf parameters. Measurements were performed in 2× and 3× fold zoom of the scanned leaf images by one operator. In total, more than 80,000 measurements with an accuracy of 0.001 cm, were collected in this analysis. The main measured morphological parameters were petiole length, leaf length, leaf width, length of the five main leaf veins on both the left and the right sides, the distance between the bases of these veins, the distances between the ends of these veins, and the angles between the veins and the central vein on both leaf sides.

Leaf index $\mathrm{LI}\;=\frac{B}{C}$ and leaf blade index $\mathrm{LBI}\;=\frac{A}{B}$, were counted, where A is leaf length, B is leaf width and C is petiole length.

The structure of the morphological variability of the leaf traits was assessed by the general variability and coefficient of determination of leaf traits [23,56,57,58,59,60,61].

The general variability is the coefficient of variability of the trait (CV) (%) counted as:

$$\mathrm{CV}=\frac{\sigma }{k}*100$$

where σ is the standard deviation and k is the mean values of the trait [23,56,57,58,59,60,61].

The average coefficient of determination of the certain trait was calculated as a square of the correlation coefficient r^2^, averaged over the entire matrix of morphological traits, as follows:

$$R_{m}^{2}=1-\frac{Y^{\prime }*\left(I-P\left(X\right)\right)*Y}{Y^{\prime }*\left(I-\pi \left(X\right)\right)*Y},$$

where X is matrix nxk-values of the factors, Y is the standard deviation, P(x) is the projection to the plane X, and π(X) is the distribution of the simple numbers [23,56,57,58,59,60,61].

The Index of Vitality of the Coenopopulation (IVC) was counted as:

$$\mathrm{IVC}=\frac{\sum_{i=1}^{N}X_{i}/{\bar{X}}_{i}}{N}$$

where X_i_ is the mean value of i-th trait for certain coenopopulation, $\bar{X}$ is the mean value of i-th trait for all coenopopulations, and N is the number of traits [23,56,57,58,59,60,61].

**Figure 6:**
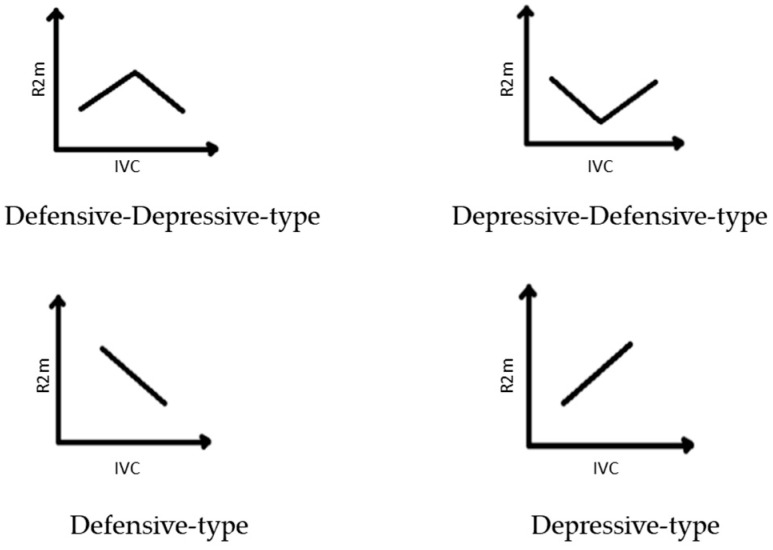
The types of graphs and wedges typical to each ontogenetic strategy.

The ontogenetic strategy of the population represents the character of the morphological integrity of plants, assessed by the coefficient of determination of traits ($R_{m}^{2}$) and by IVC, and represented by the graph wedge. Each type of strategy is characterized by its own complex of adaptive features. Evaluation of strategies was performed by the character of the graph wedge showing the changes in population response to the environment [23,56,57,58,59,60,61]. According to this, there are four different types of ontogenetic strategies (Figure 6) as follows:

1. Defensive-type strategy: the increase of stress leads to the strengthening of morphological integrity and plant development.
2. Depressive-type strategy: the increase of stress leads to the weakening of morphological integrity and plant development.
3. Defensive–depressive-type strategy: the increase of stress leads first to the strengthening and then to the weakening of morphological integrity.
4. Depressive–defensive-type strategy: the increase of stress leads first to the weakening and then to the strengthening of morphological integrity.

### 4.2. DNA Extraction and Genetic Analysis

In total, 52 trees of five distant populations (Figure 5) were included in the genetic analysis. Young and healthy leaves of each accession were collected in 2 mL tubes and dried using silica gel. The leaf material was stored at 4 °C until DNA isolation. The dried leaf material was ground and DNA extraction was performed using the CTAB protocol [62]. DNA quality was checked by agarose-gel electrophoresis and spectrophotometrically, and all samples were diluted to 20 ng µL^−1^ and stored at −20 °C.

Five ISSR [63,64] and six SCoT [41] primers were used for population analysis. PCR for the ISSR amplification was performed in a 20 μL PCR reaction mixture consisting of a 10 μL 2× HS-TaqPCR reaction buffer, including Hot Start Taq-Polymerase (Biolabmix, Novosibirsk, Russia), 0.3 μL of primer (10 µM), 1 μL of DNA (20 ng µL^−1^) and DEPC-treated water. Amplification was carried out in the MiniAmp thermal cycler (Thermo Fisher Scientific, Waltham, MA, USA) with the following program: primary denaturation for 5 min at 95 °C, then annealing during 40 cycles of 20 s at 53 °C, with elongations at 72 °C for 1 min 45 s and the final elongation at 72 °C for 7 min. The separation of ISSR fragments was performed on a 2% agarose gel for 2.5 h at 90 V in a 1 × TAE buffer. The data were recorded as a 1/0 matrix for the presence and absence of amplified fragments.

The SCoT PCR reaction mixture consisted of a 10 μL 2× HS-TaqPCR reaction buffer, including Hot Start Taq-Polymerase (Biolabmix, Novosibirsk, Russia), 0.4 μL of primer (10 µM), 2 μL of DNA (20 ng µL^−1^) and DEPC-treated water, in a total PCR volume of 20 µL. Amplification was carried out in the MiniAmp thermal cycler (Thermo Fisher Scientific, USA) with the following program: primary denaturation 5 min at 95 °C, 35 cycles with denaturation at 95 °C—1 min, annealing at 52 °C—1 min, elongation at 72 °C for 2 min and final elongation at 72 °C for 5 min. The separation of SCoT-fragments was performed in a 2% agarose gel for 2.5 h at 90 V in a 1 × TAE buffer. The data were recorded as 1/0 matrix for the presence and absence of amplified fragments.

### 4.3. Statistical Analysis

The morphological data analysis was performed using the XLSTAT tools. ANOVA was used to analyze the significance of the morphological differences with a confidence interval of 95%.

To compare the efficiency of the ISSR and SCoT makers, genetic diversity parameters were calculated using the GeneAlex ver. 6.5 software [65,66]. The analysis function ‘Matches’ in GeneAlex ver. 6.5 [67] was used to identify genotypes with identical allelic patterns within the ISSR and SCoT datasets. The online resource https://irscope.shinyapps.io/iMEC/, accessed on 15 February 2022 [68] was used to evaluate the following marker parameters:

PIC (Polymorphism information content)—probability that the marker genotype of a given offspring will allow deduction, in the absence of crossing over, and which of the two marker alleles of the affected parents it received.

D (Discriminating power)—the probability that two randomly chosen individuals have different patterns, and thus are distinguishable from one another.

Polymorphism level was also calculated. To eliminate the effect of sample number, we proposed the parameter of relative polymorphism (the ratio of *P* to *N*) for comparing the polymorphism levels of different populations.

Additionally, the XLStat software was used for hierarchical clustering, and dissimilarity was calculated using the DICE coefficient, with agglomeration by the Ward’s method. PCA analysis was used to find associations between traits. The joint matrix for these analyses contained phenotypic matrix data (33 evaluated traits) and the genetic marker data (53 polymorphic ISSR and SCoT bands) in 52 accessions of *D. lotus*. Data standardization was performed through the “Coding by ranks” function in the XLSTAT software. This coding was performed for genetic data and for morphological data, separately. After this, the results of the coding were combined in a joint matrix and analyzed by Pearson (n) PCA type.

## 5. Conclusions

Among the different morphological traits, several traits were established as more informative with a low level of general variability: leaf length, leaf width, LI and length of the fourth veins. Generally, the Depressive-type ontogenetic strategy and a low level of intraspecific genetic diversity was observed in *D. lotus* in the Northwestern Caucasus. However, a higher vitality index was observed in the northernmost population in Shkhafit, indicating a greater adaptive potential of this population, as compared to the other four populations.

The strong associations of several morphological traits (leaf length, leaf width and first to fifth right vein angles) and several marker bands (SCoT5_1250, SCot5_1500, SCoT7_850, SCoT30_800, ISSR14.1_500, ISSR14.1_770, ISSR14.1_1000, ISSR13_700, ISSR13_750, ISSR880_500 and ISSR880_550) were detected for the Shkhafit population. Based on these results, it can be concluded that most adaptations in *D. lotus* are due to both existing variation or new mutations. Unique SCoT and ISSR fragments were detected for the Shkhafit population and can be used as markers for breeding purposes. The results provide a better understanding of the adaptive mechanisms of *D. lotus* in extreme environments, and will be important for the further expansion of the cultivation area for persimmon into colder regions.

## Figures and Tables

**Table 1:** Genetic diversity parameters of ISSR and SCoT markers in *D. lotus* populations (N = 52).

Marker	Primer 5′-3′	Na	P. Bands, %	PIC	D
ISSR815	(CT)8G	10	50.00	0.35	0.13
ISSR880	(GGAGA)3	13	38.46	0.34	0.13
ISSR13	(AC)8C	7	14.29	0.33	0.27
ISSR14.1	(CT)8TG	9	44.44	0.34	0.24
Mean ± SD		9.75 ± 2.5	36.80 ± 15.7	0.34 ± 0.01	0.19 ± 0.07
SCoT05	CAACAATGGCTACCACGA	10	10.00	0.38	0.76
SCoT07	CAACAATGGCTACCACGG	4	50.00	0.46	0.35
SCoT20	ACCATGGCTACCACCGCG	12	41.67	0.39	0.63
SCoT26	ACCATGGCTACCACCGTC	9	11.11	0.39	0.75
SCoT30	CCATGGCTACCACCGGCG	11	27.27	0.43	0.61
SCoT32	CCATGGCTACCACCGCAC	6	50.00	0.39	0.64
Mean ± SD		8.67 ± 3.08	31.68 ± 18.35	0.41 ± 0.03	0.62 ± 0.15

**Table 2:** Genetic polymorphism (*P*) in different *D. lotus* populations, assessed by ISSR and SCoT markers.

POP	N	P ISSR	P SCOT	RELATIVE P ISSR	RELATIVE P SCOT
SHKHAFIT	9	7.69%	30.77%	0.85%	3.42%
GAGRA	18	46.15%	59.62%	2.56%	3.31%
SOCHI	9	17.95%	50.00%	1.99%	5.55%
SUKHUM	10	12.82%	53.85%	1.28%	5.39%
GULRIPSH	6	2.56%	32.69%	0.42%	5.45%
MEAN	10.4 ± 4.5	17.44% ± 7.62%	45.38% ± 5.79%	1.42% ± 0.11%	4.62% ± 0.15%

## Data Availability

Data are contained within the article or Supplementary Materials.

## Supplementary Materials

The following supporting information can be downloaded at: https://www.mdpi.com/article/10.3390/ijms23042192/s1.

## References

[1] Yang Y., Ruan X., Wang R. (2013). Indigenous Persimmon Germplasm Resources in China. Acta Hortic..

[2] Yildirim N., Ercisli S., Agar G., Orhan E., Hizarci Y. (2010). Genetic variation among date plum (*Diospyros lotus*) genotypes in Turkey. Genet. Mol. Res..

[3] Guan C., Zhang P., Hu C., Chachar S., Riaz A., Wang R., Yang Y. (2019). Genetic diversity, germplasm identification and population structure of *Diospyros kaki* Thunb. from different geographic regions in China using SSR markers. Sci. Hortic..

[4] Omarov M.D. (2018). Yield of different cultivars of oriental persimmon in the humid subtropics of Russia. Subtrop. Ornam. Hortic..

[5] Omarov M.D., Zagirov N.G., Omarova Z.M., Avidzba M.A., Ryndin A.V. (2014). Atlas of Cultivars and Hybrids of Oriental Persimmon.

[6] Gil-Muñoz F., Delhomme N., Quiñones A., Naval M.d.M., Badenes M.L., García-Gil M.R. (2020). Transcriptomic Analysis Reveals Salt Tolerance Mechanisms Present in Date-Plum Persimmon Rootstock (*Diospyros lotus* L.). Agronomy.

[7] Franks S.J., Weber J.J., Aitken S.N. (2014). Evolutionary and plastic responses to climate change in terrestrial plant populations. Evol. Appl..

[8] Radersma R., Noble D.W.A., Uller T. (2020). Plasticity leaves a phenotypic signature during local adaptation. Evol. Lett..

[9] Ramírez-Valiente J.A., Sánchez-Gómez D., Aranda I., Valladares F. (2010). Phenotypic plasticity and local adaptation in leaf ecophysiological traits of 13 contrasting cork oak populations under different water availabilities. Tree Physiol..

[10] Engel K., Tollrian R., Jeschke J.M. (2011). Integrating biological invasions, climate change and phenotypic plasticity. Commun. Integ. Biol..

[11] Henn J.J., Buzzard B., Enquist B.J., Halbritter A.H., Klanderud K., Maitner B.S., Michaletz S.T., Pötsch C., Seltzer L., Telford R.J. (2018). Intraspecific Trait Variation and Phenotypic Plasticity Mediate Alpine Plant Species Response to Climate Change. Front. Plant Sci..

[12] Wang M., Zhang J., Guo Z., Guan Y., Qu G., Liu J., Guo Y., Yan X. (2020). Morphological variation in Cynodon dactylon (L.) Pers., and its relationship with the environment along a longitudinal gradient. Hereditas.

[13] Arantes M.K., da Silva Filho M.P., Pennacchi J.P., Mendonca A.M.C., Barbosa J.P.R.A.D. (2020). Phenotypic plasticity of leaf anatomical traits helps to explain gas-exchange response to water shortage in grasses of different photosynthetic types. Theor. Exp. Plant Physiol..

[14] Nicotra A.B., Atkin O.K., Bonser S.P., Davidson A.M., Finnegan E.J., Mathesius U., Poot P., Purugganan M.D., Richards C.L., Valladares F. (2010). Plant phenotypic plasticity in a changing climate. Trends Plant Sci..

[15] Liu Y., El-Kassaby Y.A. (2019). Phenotypic plasticity of natural Populus trichocarpa populations in response to temporally environmental change in a common garden. BMC Evol. Biol..

[16] Gentili R., Ambrosini R., Augustinus B.A., Caronni S., Cardarelli E., Montagnani C., Müller-Schärer H., Schaffner U., Citterio S. (2021). High Phenotypic Plasticity in a Prominent Plant Invader along Altitudinal and Temperature Gradients. Plants.

[17] Alberto F.J., Aitken S.N., Ali R., Gonzalez-Martinez S.C., Hänninenk H., Kremer A., Lefèvre F., Lenormand T., Yeaman S., Whetten R. (2013). Potential for evolutionary responses to climate change—Evidence from tree populations. Glob. Change Biol..

[18] Rostova N.S. (2002). Correlations: Structure and Variability.

[19] Gratani L. (2014). Article Plant Phenotypic Plasticity in Response to Environmental Factors Review. Adv. Bot..

[20] Pérez-Ramos I.M., Matías L., Gómez-Aparicio L., Godoy Ó. (2019). Functional traits and phenotypic plasticity modulate species coexistence across contrasting climatic conditions. Nat. Commun..

[21] Niinemets U. (2020). Leaf Trait Plasticity and Evolution in Different Plant Functional Types. Annu. Plant Rev..

[22] Flury B.N. (1988). Common Principal Components and Related Multivariate Models.

[23] Ishbirdin A.R., Ishmuratova M.M., Zhirnova T.V. (2005). Life strategies of cenopopulation *Cephalanthera rubra* (L.) Rich. on the territory of the Bashkir State Reserve. Bull. Nizhny Novgorod Univ. N.I. Lobachevsky Ser. Biol..

[24] Le Corre V., Kremer A. (2012). The genetic differentiation at quantitative trait loci under local adaptation. Mol. Ecol..

[25] Andrews C.A. (2010). Natural Selection, Genetic Drift, and Gene Flow Do Not Act in Isolation in Natural Populations. Nat. Educ. Knowl..

[26] Raddová J., Ptáčková H., Čechová J., Ondrášek I. (2012). Genetic analysis of the genus *Diospyros* ssp. using RAPD and i-PBS methods. Acta Univ. Agric. Silvic. Mendel. Brun..

[27] Liang Y., Han W., Sun P., Liang J., Wuyun T., Li F., Fu J. (2015). Genetic diversity among germplasms of *Diospyros kaki* based on SSR markers. Sci. Hortic..

[28] Guo D.-L., Luo Z.R. (2011). Genetic relationships of the Japanese persimmon *Diospyros kaki* (*Ebenaceae*) and related species revealed by SSR analysis. Genet. Mol. Res..

[29] Pinar H., Yildiz E., Kaplankiran M., Toplu C., Unlu M., Serce S., Ercisli S. (2017). Molecular characterization of some selected persimmon genotypes and cultivars by srap and ssr markers. Genetika.

[30] Soriano J.M., Pecchioli S., Romero C., Vilanova S., Llacer G., Giordani E., Badenes M.L. (2006). Development of microsatellite markers in polyploid persimmon (*Diospyros kaki* L.) from an enriched genomic library. Mol. Ecol. Notes.

[31] Naval M., Zuriaga E., Pecchioli S., Llácer G., Giordani E., Badenes M.L. (2010). Analysis of genetic diversity among persimmon cultivars using microsatellite markers. Tree Genet. Genomes.

[32] Yonemori K., Honsho C., Kanzaki S., Ino H., Ikegami A., Kitajima A., Sugiura A., Parfitt D.E. (2007). Sequence analyses of the ITS regions and the matK gene for determining phylogenetic relationships of *Diospyros kaki* (persimmon) with other wild *Diospyros* (*Ebenaceae*) species. Tree Genet. Genomes.

[33] Jing Z., Ruan X., Wang R., Yang Y. (2013). Genetic diversity and relationships between and within persimmon (*Diospyros* L.) wild species and cultivated varieties by SRAP markers. Plant. Syst. Evol..

[34] Guan C., Chachar S., Zhang P., Hu C., Wang R., Yang Y. (2020). Inter- and Intra-specific Genetic Diversity in *Diospyros* Using SCoT and IRAP Markers. Hortic. Plant J..

[35] Deng L., Liang Q., He X., Luo C., Chen H., Qin Z. (2015). Investigation and Analysis of Genetic Diversity of *Diospyros* Germplasms Using SCoT Molecular Markers in Guangxi. PLoS ONE.

[36] Yang Y., Yang T., Jing Z. (2015). Genetic diversity and taxonomic studies of date plum (*Diospyros lotus* L.) using morphological traits and SCoT markers. Biochem. Syst. Ecol..

[37] Samarina L.S., Malyarovskaya V.I., Reim S., Koninskaya N.G., Matskiv A.O., Tsaturyan G.A., Rakhmangulov R.S., Shkhalakhova R.M., Shurkina E.S., Kulyan R.V. (2021). Genetic Diversity in Diospyros Germplasm in the Western Caucasus Based on SSR and ISSR Polymorphism. Biology.

[38] Fu J., Liu H., Hu J., Liang Y., Liang J., Wuyun T., Tan X. (2016). Five Complete Chloroplast Genome Sequences from *Diospyros*: Genome Organization and Comparative Analysis. PLoS ONE.

[39] Reddy P.M., Sarla N., Siddiq E. (2002). Inter simple sequence repeat (ISSR) polymorphism and its application in plant breeding. Euphytica.

[40] Azhar M., Muhammad H.A., Siti N.I., Parween K.S.A.S. (2013). Optimization of ISSR Markers for Molecular DNA Fingerprinting in Aquilaria sp. Nuclear Technical Convention.

[41] Collard B.C.Y., Mackill D.J. (2009). Start Codon Targeted (SCoT) Polymorphism: A simple, novel DNA marker technique for generating gene-targeted markers in plants. Plant Mol. Biol. Rep..

[42] Etminan A., Pour-Aboughadareh A., Mohammadi R., Ahmadi-Rad A., Noori A., Mahdavian Z., Moradi Z. (2016). Applicability of start codon targeted (SCoT) and inter-simple sequence repeat (ISSR) markers for genetic diversity analysis in durum wheat genotypes. Biotechnol. Biotechnol. Equip..

[43] Robert H.G., Faik A.A., Millson M., Huang H.S., Chuang L.T., Sanz C., Golding J.B. (2005). Changes in sugars, acids and fatty acids in naturally parthenocarpic date plum persimmon (*Diospyros lotus* L.) fruit during maturation and ripening. Eur. Food Res. Technol..

[44] Conner J.K., Hartl D.L. (2004). A Primer of Ecological Genetics.

[45] Rubio De Casas R., Vargas P., Pe’rez-Corona E., Manrique E., Quintana J., Garcı’a-Verdugo C., Balaguer L. (2007). Field patterns of leaf plasticity in adults of the long-lived evergreen Quercus coccifera. Ann. Bot..

[46] Rehfeldt G.E. (1991). A model of genetic variation for Pinus ponderosa in the inland northwest (USA): Applications in gene resource management. Can. J. Res..

[47] Gorji A.M., Poczai P., Polgar Z., Taller J. (2011). Efficiency of arbitrarily amplified dominant markers (SCoT, ISSR and RAPD) for diagnostic fingerprinting in tetraploid potato. Am. J. Potato Res..

[48] Zeng B., Zhang Y., Huang L.K., Jiang X.M., Luo D., Yin G.H. (2014). Genetic diversity of orchardgrass (*Dactylis glomerata* L.) germplasms with resistance to rust diseases revealed by StartCodon Targeted (SCoT) markers. Biochem. Syst. Ecol..

[49] Kumar J., Agrawal V. (2019). Assessment of genetic diversity, population structure and sex identification in dioecious crop, Trichosanthes dioica employing ISSR, SCoT and SRAP markers. Heliyon.

[50] Samarina L.S., Matskiv A.O., Koninskaya N.G., Shkhalakhova R.M., Gvasaliya M.V., Tsaturyan G.A., Ryndin A.V., Pchikhachev E.K., Manakhova K.A., Shumeev A.N. (2022). Genetic diversity and genome size variability in core collection of tea plant (*Camellia sinensis* L. Kuntze) in Russia. Front. Plant Sci..

[51] Samarina L.S., Malyarovskaya V.I., Reim S., Yakushina L.G., Koninskaya N.G., Klemeshova K.V., Shkhalakhova R.M., Matskiv A.O., Shurkina E.S., Gabueva T.Y. (2021). Transferability of ISSR, SCoT and SSR Markers for Chrysanthemum × Morifolium Ramat and Genetic Relationships among Commercial Russian Cultivars. Plants.

[52] Stojnić S., Avramidou E.V., Fussi B., Westergren M., Orlović S., Matović B., Trudić B., Kraigher H., Aravanopoulos F.A., Konnert M. (2019). Assessment of genetic diversity and population genetic structure of norway spruce (*Picea abies* (L.) Karsten) at its southern Lineage in Europe. Implications for conservation of forest genetic resources. Forests.

[53] Konopiński M.K. (2020). Shannon diversity index: A call to replace the original Shannon’s formula with unbiased estimator in the population genetics studies. PeerJ.

[54] Abramoff M.D., Magalhães P., Ram S.J. (2004). Image Processing with Image. J. Biophotonics Int..

[55] Nouri A., Golabadi M., Etminan A., Rezaei A., Mehrabi A. (2021). Comparative assessment of SCoT and ISSR markers for analysis of genetic diversity and population structure in some *Aegilops tauschii* Coss. accessions. Plant Genet. Resour. Charact. Util..

[56] Bogoslov A.V., Kashin A.S., Parkhomenko A.S., Kulikova L.V., Shilova I.V., Knjazeva A.K. (2021). Vitality Structure of *Colchicum bulbocodium* subsp. *versicolor* (*Colchicaceae, Liliopsida*) Populations in the Lower Volga Region. Biol Bull. Russ. Acad. Sci..

[57] Kashin A.S., Petrova N.A., Shilova I.V. (2016). Some features of the environmental strategy of *Tulipa gesneriana* L. (*Liliaceae*, *Liliopsida*). Povolzhskiy Ekol. Zhurnal..

[58] Shabanov D.A., Korshunov A.V., Kravchenko M.A., Meleshko E.V., Shabanova A.V., Usova E.E. (2014). The intrapopulation developmental strategies of precocity and stuntedity: Determination by the example of anurans. J. V.N. Karazin Kharkiv Natl. Univ..

[59] Zlobin Y., Kovalenko I., Klymenko H., Kyrylchuk K., Bondarieva L., Tykhonova O., Zubtsova I. (2021). Vitality Analysis Algorithm in the Study of Plant Individuals and Populations. Open Agric. J..

[60] Zlobin Y.A. (1989). Principles and Methods of Studying Coenopopulations.

[61] Ishbirdin A.R., Ishmuratova M.M. (2004). Adaptive morphogenesis and ecological-coenotic strategies for the survival of herbaceous plants. Methods of Population Biology: Materials of VII All-Russian.

[62] Doyle J.J., Doyle J.L. (1991). Isolation of plant DNA from fresh tissue. Focus.

[63] Mondal T.K. (2002). Assessment of genetic diversity of tea (*Camellia sinensis* (L.) O. Kuntze) by inter-simple sequence repeat polymerase chain reaction. Euphytica.

[64] Roy S.C., Chakraborty B.N. (2009). Genetic diversity and relationsips among tea (*Camellia sinensis*) cultivars revealed by RAPD and ISSR based fingerprinting. Indian J. Biotechnol..

[65] Peakall R., Smouse P.E. (2006). GENALEX 6: Genetic analysis in Excel. Population genetic software for teaching and research. Mol. Ecol. Notes.

[66] Peakall R., Smouse P.E. (2012). GenAlEx 6.5: Genetic analysis in Excel. Population genetic software for teaching and research—An update. Bioinformatics.

[67] Pritchard J.K., Stephens M., Donnelly P. (2000). Inference of population structure using multilocus genotype data. Genetics.

[68] Amiryousefi A., Hyvönen J., Poczai P. (2018). iMEC: Online Marker Efficiency Calculator. Appl. Plant Sci..

